# Applying the COM-B model to creation of an IT-enabled health coaching and resource linkage program for low-income Latina moms with recent gestational diabetes: the STAR MAMA program

**DOI:** 10.1186/s13012-016-0426-2

**Published:** 2016-05-18

**Authors:** Margaret A. Handley, Elizabeth Harleman, Enrique Gonzalez-Mendez, Naomi E. Stotland, Priyanka Althavale, Lawrence Fisher, Diana Martinez, Jocelyn Ko, Isabel Sausjord, Christina Rios

**Affiliations:** 1 Department of Epidemiology and Biostatistics, University of California San Francisco, San Francisco, USA; 2 Division of General Internal Medicine, UCSF/Zuckerberg San Francisco General Hospital, University of California San Francisco, San Francisco, USA; 3 UCSF Center for Vulnerable Populations at Zuckerberg San Francisco General Hospital, San Francisco, CA 94110 USA; 4 Department of Obstetrics and Gynecology, University of California San Francisco, San Francisco, USA; 5 Zuckerberg San Francisco General Hospital, San Francisco, USA; 6 Department of Family and Community Medicine, University of California San Francisco, San Francisco, USA; 7 Vista Family Health Center, Santa Rosa, USA; 8 University of California San Francisco, San Francisco, USA

**Keywords:** COM-B model, Behavior change theory, Diabetes prevention, Diabetes Prevention Program, Health disparities

## Abstract

**Background:**

One of the fastest growing risk groups for early onset of diabetes is women with a recent pregnancy complicated by gestational diabetes, and for this group, Latinas are the largest at-risk group in the USA. Although evidence-based interventions, such as the Diabetes Prevention Program (DPP), which focuses on low-cost changes in eating, physical activity and weight management can lower diabetes risk and delay onset, these programs have yet to be tailored to postpartum Latina women. This study aims to tailor a IT-enabled health communication program to promote DPP-concordant behavior change among postpartum Latina women with recent gestational diabetes.

The COM-B model (incorporating Capability, Opportunity, and Motivational behavioral barriers and enablers) and the Behavior Change Wheel (BCW) framework, convey a theoretically based approach for intervention development. We combined a health literacy-tailored health IT tool for reaching ethnic minority patients with diabetes with a BCW-based approach to develop a health coaching intervention targeted to postpartum Latina women with recent gestational diabetes. Current evidence, four focus groups (*n* = 22 participants), and input from a Regional Consortium of health care providers, diabetes experts, and health literacy practitioners informed the intervention development. Thematic analysis of focus group data used the COM-B model to determine content. Relevant cultural, theoretical, and technological components that underpin the design and development of the intervention were selected using the BCW framework.

**Results:**

STAR MAMA delivers DPP content in Spanish and English using health communication strategies to: (1) validate the emotions and experiences postpartum women struggle with; (2) encourage integration of prevention strategies into family life through mothers becoming intergenerational custodians of health; and (3) increase social and material supports through referral to social networks, health coaches, and community resources. Feasibility, acceptability, and health-related outcomes (weight loss, physical activity, consumption of healthy foods, breastfeeding, and glucose screening) will be evaluated at 9 months postpartum using a randomized controlled trial design.

**Conclusions:**

STAR MAMA provides a DPP-based intervention that integrates theory-based design steps. Through systematic use of behavioral theory to inform intervention development, STAR MAMA may represent a strategy to develop health IT intervention tools to meet the needs of diverse populations.

**Trial registration:**

ClinicalTrials.gov NCT02240420

**Electronic supplementary material:**

The online version of this article (doi:10.1186/s13012-016-0426-2) contains supplementary material, which is available to authorized users.

## Background

Gestational diabetes mellitus (GDM) is defined as diabetes diagnosed in and confined to pregnancy [1]. The incidence of GDM is increasing and now affects 7–10 % of US pregnancies [2, 3]. GDM leads to short-term morbidity but also serves as a sentinel event in the lives of reproductive-aged women that indicates risk of future glucose intolerance, placing women at high risk for type-2 diabetes mellitus (T2DM) and cardiovascular disease, the leading cause of death among US women [4, 5]. GDM is one of the strongest predictors of development of T2DM, and women with a GDM pregnancy represent the single most identifiable risk group for subsequent rapid onset of T2DM among younger-aged populations [1, 3, 4]. A 2009 systematic review concluded that women with prior GDM (pGDM) have seven times the risk of developing T2DM relative to women with normo-glycemic pregnancies, with the most risk conferred within 5 years of delivery [3]. Furthermore, the effect of GDM spans multiple generations by influencing metabolic programming in a woman’s offspring [6]. In the USA, Latina and Asian women and women born outside of the USA have the highest GDM rates [7]. A recent study examining trends in hospital deliveries for GDM-complicated pregnancies found that Hispanics had the largest increase in GDM prevalence, with a 66 % increase between 2000 and 2010 [8]. In California, Latina women represent the largest number of GDM patients [9].

The diabetes prevention literature indicates that the long-term risk for developing T2DM can be reduced significantly through behavior change related to diet, exercise, and metformin therapy, with diet and physical activity conferring the most potent risk reduction [3, 10–12]. This seminal evidence comes from the Diabetes Prevention Program (DPP) trial which found that compared to control the incidence of T2DM was reduced by 58 % with the lifestyle intervention and 31 % with metformin [13, 14]. There were too few Hispanic and Asian participants to examine ethnic subgroup differences in outcomes, but the overall findings of significant risk reduction through diet and exercise changes have resulted in significant efforts to take the DPP to scale.

Consequently, modified versions of the DPP have been translated into a variety of clinic and community-based programs, but few focus specifically on pGDM women [15, 16]. One recent study using DPP curriculum for pGDM women found that among English-speaking pGDM women in a large Health Maintenance Organization (Kaiser), those receiving in-person and telephone counseling were more likely to reach pre-pregnancy weight goals than women not receiving the counseling [17]. Unfortunately, there are no other existing versions of DPP-adapted programs for pGDM women, and there are no data to suggest which components need to be tailored to the diverse needs of pGDM women, for example, women who are non-English speakers or women with migration history. Furthermore, although there are many evidence-based recommendations for preventing diabetes through lifestyle-based interventions for at-risk adults, for the pGDM population specifically, these recommendations focus on receipt of timely postpartum T2DM screening and not on encouraging the uptake of lifestyle-based prevention activities after delivery [18, 19].

Health information technology (HIT) can be an important tool to tailor health communication efforts and, if developed with end-users, can be particularly effective with low literacy and low-income populations. However, as many telephone data plans are priced by the data minute, they may prohibit engaging in lifestyle-focused apps that could be used for diabetes prevention in pGDM women. This is substantiated by the Pew Report (2015), which indicates that 85 % of all US adults have a cell phone, while only 53 % use a smart-phone. Recent data among Latinos (surveyed in Spanish through the Pew Internet and American Life Project) suggest that the lowest internet use rates were among foreign-born Latinos, those with lower education, those having incomes below US $ 30,000 a year, and those living in rural areas [20]. Automated Telephone Self-Management Support (ATSM) is defined as automated telephone messages that provide tailored education, tips, or resource links in the form of short narratives and texts, and that engage participants to report behavior through queries that are fed back to a health coach for ‘live’ follow-up. ATSM programs represent a middle ground between live counseling and internet-based apps for tailored health communication and can access hard to reach populations not engaged in visit-based programs. ATSM programs also provide tailored pre-programmed content, yet can also be linked to trigger a ‘live’ health coach call back to discuss selected topics. ATSM programs have been particularly successful in reaching and engaging patients with low health literacy and limited English proficiency in the area of diabetes self-management [21–27] but have not been applied to diabetes prevention.

This paper describes the theoretical framework and development process for *STAR MAMA* (“*STAR MAMA*” [*S*upport via *T*elephone *A*dvice and *R*esources/*S*istema *Telefónico * de *A*poyo y *R*ecoursos-MAMA)]. STAR MAMA is a bilingual (English and Spanish) postpartum diabetes prevention ATSM-based health coaching program designed to decrease diabetes risk behaviors among pGDM women in the San Francisco Bay Area. STAR MAMA is focused specifically on implementation of an adapted version of the DPP among primarily younger pGDM Latina women (ages 18–39 years). An ATSM model was selected at the outset because it allows participants to receive content and health coaching support in their primary language on a weekly basis while remaining in their homes, as traveling to appointments and group sessions are known to be an important barrier to receiving preventive services in community settings. We used a theory-informed step-wise approach based on the recently developed COM-B model and Behaviour Change Wheel (BCW) to adapt the DPP to a high-risk population for type 2 diabetes. To our knowledge, the COM-B model and BCW have not been previously applied to adapting diabetes prevention interventions for high-risk postpartum women.

## Methods

### Theoretical approach to the development of the STAR MAMA intervention for pGDM women

There are many ways to include behavior change theory in order to understand what to target for interventions and guide selection of a “coordinated set of activities” to help plan out intervention development. However, as others have pointed out, the majority of the individual theories and models of behavior change do not include a strong emphasis on the context in which a behavior occurs, often fail to focus on reflective processes that affect behavior (eg. beliefs and feelings), and are unable to explicitly state how to bring about change [28–30]*.* Fortunately, in recent years, synthesis reviews of successful behavior change interventions have produced a greater understanding of the theoretical constructs associated with successful behavior change in a variety of settings [30].

We have selected the Behavior Change Wheel (BCW) as a guiding framework for the development of the STAR MAMA program. The BCW was developed from a synthesis of 19 frameworks of behavior change in a systematic literature review [30]. It includes three inter-related layers, the first uses the Capability, Opportunity and Motivation model (COM-B model) to help identify the sources of behavior that may be selected for intervention targets [30–34]. The second layer of the BCW provides direction to help identify intervention options, by a process of selecting from among nine intervention functions that could be applied to an intervention to address the behavioral barriers and leverage potential enablers identified with the COM-B analysis [31]. The outer layer of the BCW identifies seven policy options that can be employed to help deliver the intervention functions selected. These include as follows: service provision, communication/marketing, fiscal measures, regulation, guidelines, legislation, and environmental/social planning.

The Theoretical Domains Framework (TDF) is related to the BCW and provides a synthesis of constructs derived from behavior change theories that was developed in a consensus process [30]. The 12 domains of the TDF include knowledge; social role and identity; emotion; skills; beliefs about capabilities, self-efficacy; environmental context and resources; beliefs about consequences; memory, attention and decision processes; behavioral regulation; social influences; motivation and goals; and the nature of the behavior [33]. Each domain has a set of theoretical constructs that relate to it that comes from existing theories of behavior change [35]. Because each domain of the TDF relates to a COM-B component, and using the two together allows for an expansion of the COM-B components into highly specific domains, we also used the TDF to guide intervention development [33].

The COM-B model organizes domains into capability, opportunity, and motivation-related factors: *Capability* refers to the ability to engage in the thoughts or physical processes necessary for the behavior and includes *both psychological and physical capability*; *opportunity* refers to factors in the environment or social setting that influence behavior and includes social and physical opportunity; and *motivation* refers to beliefs and emotions/impulses that are not always consciously recognized, but often direct behavior, and encompasses reflective and automatic motivation [30, 33]. Through a behavioral diagnosis phase, a problem is understood in terms of capability, opportunity, and motivational barriers and enablers. Then, after the specific barriers and enablers are identified, using the BCW helps organize and select which functions of an intervention could ‘map’ to the barrier (for example, intervention components such as education, skill building, persuasion). Following selection of intervention functions, the final step is to determine how you want to deliver the component, for example, how you might deliver education (such as 1:1 counseling, health coaching, training). Taken together, this approach can link: (1) a comprehensive assessment of potential barriers to behavior change in a particular setting; (2) a rational process for selecting which barriers to target; and (3) a method to match evidence-based strategies for overcoming barriers to those that have been identified and prioritized.

We used the COM-B model and a step-wise process for developing STAR MAMA. The steps in the intervention development process are adapted from *French 2012* [35] and *Michie et al.* [30–33] and summarized in Table 1 *.*

**Table 1:** Development steps for adaptation of DPP content to pGDM Latina women

Step	Activities and inputs	Message design outcomes	Tailoring for STAR MAMA population
Step 1: Characterizing the evidence-practice gap regarding diabetes prevention behaviors that are essential components of the DPP, for pGDM women and their families	Regional Consortium (RC) review of DPP curriculum and other intervention literature to identify: 1. diabetes prevention topics a most relevant to pGDM women and 2. potential health coaching delivery methods.	DPP curriculum selected targeting physical activity, stress relief, and healthy eating, with added emphasis on breastfeeding, infant care, mental health, family centered and peer-based social support, and health literacy skill building.	Message framing emphasizes emotional truths encountered for women, especially in the context of migration: e.g., the positive role of mothers as intergenerational custodians of family health, and the stress and sorrow of social isolation and challenges in reaching out to others for help.
	Conduct of focus groups (FG) 1 and 2 to identify barriers and enablers among pGDM women about diabetes prevention activities in both rural and urban settings.		
Step 2: Understanding barriers and enablers for diabetes prevention among postpartum women	RC review of FG data and pros and cons of different health IT technologies, such as ATSM, texting, or radionovellas.	ATSM-text blended model selected (vs text alone) with weekly format.	ATSM calls adapted in Spanish and English and calls dispatched at participant’s preferred times.
Step 3: Identifying which barriers and enablers need to be addressed	Analysis of FG data and mapping of theoretical constructs from TDF/COM-B relevant to identified barriers and enablers.	Added narratives addressing low risk perceptions, limited ability to leave house for exercise, tips for understanding nutrition and labels and on eliciting partner support in family health.	Prioritization of skill building around nutrition, and detailed examples of how women achieved successes for their family. Women’s preferences reflected 4–5 minute weekly calls with questions, narratives, and texting opt in tips.
	Creation of recorded prototype behavioral questions, narratives, and texting examples to elicit reactions in FG 3–4 and identify additional content.		
Step 4: Determining which intervention components, including behavior change techniques and modes of delivery, could overcome the modifiable barriers identified, and enhance the enablers	Final selection of content and frequency and duration of STAR MAMA based on barriers identified through FG 3–4 and enablers suggested by participants.	Delivery of behavior change support, diabetes prevention messages, and educational health coaching through the ATSM model.	Adaptations to narratives, queries and texts within the ATSM model to account for family values, key challenges (such as community influencers) and the desire to maintain cultural traditions while balancing a healthy lifestyle.
Step 5: Determining which policy categories could help encourage STAR MAMA content to be delivered in targeted settings	Creation of health coaching training materials to deliver to partner organizations involved in STAR MAMA, primarily WIC nutritionists who would be delivering health coaching call backs to STAR MAMA participants (service delivery).	For each of the weeks and for each of the queries and narratives included in STAR MAMA calls, there was a companion health coaching guide for use in call backs.	The RC reviewed these for acceptability to partners involved in STAR MAMA, primarily clinic staff at the primary care, high-risk obstetrics, and WIC programs that were involved in the pilot.

Step 1. Characterize the evidence-practice gap regarding diabetes prevention behaviors that are essential components of the DPP.

This first step involves understanding a problem in terms of the evidence needing to be translated. In this case, the problem is related to the challenges pGDM women face to meet DPP recommendations for diabetes prevention (What evidence-based components of the DPP need to be translated to improve diabetes prevention behaviors among pGDM women, and in particular pGDM Latina women and their families?).

Step 2. Understanding barriers and enablers that affect key behaviors involved in overcoming the evidence-practice gap being translated. This relates to understanding barriers and enablers to diabetes prevention among postpartum women, specifically, for behaviors related to weight loss, dietary changes, breastfeeding and physical activity among pGDM women (What are the barriers and enablers for DPP-relevant diabetes prevention behaviors experienced by pGDM women, which occur at the individual, family, and community level, across generations, and in the larger context of macroeconomic forces, such as migration?).

Step 3. Identifying which barriers and enablers should be addressed. Specifically, this involves understanding barriers and enablers in terms of theoretical constructs that can be targeted for aiding in behavior change, such as capability, opportunity, and motivation (Drawing from theoretical frameworks, which modifiable behavioral and non-behavioral barriers and enablers need to be addressed in the intervention components?).

We conducted a series of four focus groups with postpartum Latina women with either a recent pregnancy complicated by GDM history or a recent BMI >25. The principal goals of the focus groups were to: (1) identify individual, family-level, and contextual behavioral and non-behavioral barriers and enablers experienced by postpartum women and (2) identify ‘*emotional truths*’ that women conveyed during their discussions of barriers they encountered. Emotional truths are insights or observations that resonate affectively with a target audience and influence decision-making [36]. When used in message development they can increase attention to messages when they are first heard, help recall the messages at a later time, and impact and change attitudes [36]. In the context of creating the STAR MAMA program, we wanted to hear from women in the groups not just ‘the facts’ of the barriers they encountered but also about how the barrier shaped their experiences. For example, “*I am not able to prepare healthy meals as often as I want to because my children don’t enjoy vegetables and complain when they are not allowed to have soda*” (P2-FG3), would convey a barrier to healthy eating, but understanding the emotional impact on her requires exploring the feelings this creates, in this case, about providing satisfying foods to her children (e.g., “*I am not happy because I want to be a mother that provides enjoyable meals, as I feel my mother did, and I am not doing that when I say ‘no’ to sodas at dinner*,” P3-FG3). Through understanding emotional truths experienced by focus group participants, we were able to turn composites of these experiences into first-person narratives in STAR MAMA.

### Focus groups—methods

Focus groups 1 and 2 were done concurrently with Step 2. Between June 2011 and April 2013, we conducted four focus groups with postpartum women (three in Spanish and one in English) for a total of 22 women in two geographic areas with high rates of GDM and that reflected both urban (San Francisco) and rural (Sonoma County) environments. Although two participants were not Latina and were included as part of an ongoing postpartum group for pGDM women, the majority of women were (90 %), most of whom (88 %) had migrated to the USA from Mexico and Central America. Many of the 22 Latina participants (86 %) reported ‘low’ or ‘some’ risk for T2DM. The mean age was 31.5 years and all but two women spoke Spanish in the home. The focus group recruitment was based on convenience samples using site-based registries of women at two primary health clinics and two Health Department-run Women, Infant, and Children (WIC) sites, with database confirmation of a recent GDM pregnancy (up to 2 years prior) or high postpartum BMI (>25), followed by recruitment letters and telephone follow-up to identify eligible women. Focus groups were moderated by bilingual and bicultural staff and were audiotaped and then transcribed and reviewed by the research team and Regional Consortium (RC). All women who were initially contacted agreed to the study although not all were able to attend the scheduled focus group sessions. The focus group study was approved by the UCSF Committee on Human Research. Women received a gift voucher worth $25 for participating.

During the groups, we explored motivational barriers, such as beliefs about the benefits of avoiding diabetes for themselves and their families and self-efficacy to change their eating habits; capability barriers, such as whether women had skills related to self-monitoring their diet and exercise; and opportunity barriers, such as asking about social norms and influence of peers on behaviors, and values placed on social support. In addition, we included physical opportunity-related questions to investigate environmental factors that can help or interfere with prevention efforts (such as economic barriers, physical environment, and food insecurity).

We also explored *health literacy-specific* barriers related to an individual’s ability to specify planned health actions and to identify environmental cues to actions, and because recent studies have identified that incorporating culture-specific values can significantly improve interventions targeted at health behavior change among Latinos, we also incorporated additional probes to responses that touched on topics such as *familismo* (family orientation), *personalismo* (preference for relationships with individuals not institutions), *fatalismo* (fatalism), *respeto* (respect), *confianza* (relationship trust), and *aguantarse* (the ability to withstand stressful situations).

The focus group guide included a series of open-ended questions for the following topics: dietary changes, reducing sedentary behaviors, weight loss, breastfeeding, preventive health care, and stress management, with probes related to what was ‘behind’ the barriers and facilitators they described, using the theoretical domains. For example, women were asked to discuss barriers to healthy eating in the context of their roles in their family, the presence or absence of social support, and the influences of migration, which have been shown to have a large impact on interventions that aim to improve women’s ability to adopt healthy behaviors such as physical activity [37, 38]. These additional probes often brought to light the larger emotions behind the barriers, for example, if a woman described a lack of physical activity opportunities in the neighborhood, the underlying associated emotions such as isolation and loneliness were explored through follow-up questions as to how women felt about the barrier.

Data analysis to identify diabetes-relevant barriers and facilitators was based on qualitative data analysis methods described by Sandelowski and others [39, 40], which we have used previously [41] in which general topic area prompts are used to orient qualitative data descriptions. In the analysis, we used the COM-B model and TDF to categorize barriers in terms of capability-, opportunity-, and motivation-related barriers or multiple barriers. In addition to discussing challenges to diabetes prevention-related behaviors, women in focus groups 2–4 also were asked to evaluate narratives that had been developed after the first focus group, for resonance and acceptability, for which suggested modifications were then incorporated into the final STAR MAMA topics. Focus group findings were discussed in a series of meetings among members of the STAR MAMA RC.

Step 4. Determining which intervention components, including content and modes of delivery, could overcome the modifiable barriers identified and enhance the enablers. This step focuses on linking the behaviors to specific intervention functions and delivery strategies, such as modeling healthy eating behaviors or providing training on stress management through exercise or relaxation (Which intervention components, including behavior change techniques and modes of delivery, could overcome the modifiable barriers identified and enhance the enablers?)*.*

Step 5. Determining which policy categories from the BCW (which include a wide range, such as delivering services or conducting communication campaigns, as well as environmental planning, establishing new guidelines, fiscal policies, regulations, or legislation) could help encourage STAR MAMA content to be delivered in targeted settings. This step involves mapping the selected intervention functions to policy categories (Which policy categories could help encourage STAR MAMA content to be delivered in targeted settings?).

## Results

### Step 1. Characterize the evidence-practice gap regarding diabetes prevention behaviors that are essential components of the DPP, for pGDM women, and their families—who needs to do what differently to achieve weight loss, improved nutrition, and increased amounts of physical activity?

The first step to creating the STAR MAMA intervention was to review the existing DPP curriculum (in the form of outlines, training guides, and individual action plan materials), and identify content areas for inclusion as topics for STAR MAMA. DPP materials focus on activities directed at achieving the following outcomes: (1) weight loss (e.g., 7 % as a goal) through a combination of physical activity and healthy eating; (2) dietary changes to increase intake of fruits and vegetables; (3) reductions in sugars, carbohydrates, and fats (substitution of less fatty foods, portion control; drinking water instead of sodas); (4) increased physical activity (e.g., walking an average of 30 min a day most days for 150 min a week, and reducing sedentary activities); (5) glucose screening testing according to relevant guidelines; and (6) stress management/well-being related activities. DPP curriculum for each of these outcomes was reviewed and discussed over a series of several meetings with the STAR MAMA RC (see Acknowledgements for a list of RC members). RC members also reviewed focus group findings for understanding behaviors. The selected DPP curriculum topic areas and related adapted STAR MAMA content areas are provided in Table 2.

**Table 2:** DPP curriculum topics selected for STAR MAMA queries, narratives, and health coaching

DPP curriculum topics	DPP topic selected for STAR MAMA
I. Physical Activity ° Getting started with Being Active (1B) ° Move Those Muscles (2 or 5) ° Being Active: A Way of Life (1A) ° Jump Start Your Activity Plan (13)	I. Physical Activity ° Promoting a first goal of starting to be physically active ° Working up to being physically active 150 min/week ° Planning adding physical activity to daily life in small steps ° Finding enjoyable physical activities and varying them
II. Nutrition ° Be a Fat Detective (Section 4 or 2) ° Ways to Eat Less Fat (6 or 4, 8) ° Healthy Eating (8) ° Tip the Calorie Balance (8) ° 4 Keys to Healthy Eating Out (10) ° The Slippery Slope of Lifestyle Change (12) ° Make Social Cues Work for You (14)	II. Nutrition ° Making healthier food choices- understanding substitutions, portions, high fat foods, labels, organic foods ° Reducing meat consumption ° Understanding different types of carbohydrates ° Reducing sweets, junk food, sugary drinks ° Strategies to eat healthy while eating out, or in social settings ° Action planning around nutrition changes ° Combatting cues leading to stress-eating
III. Mental Health and Stress ° Problem Solving (9) ° Talk Back to Negative Thoughts (11) ° You Can Manage Stress (15) ° Ways to Stay Motivated (16) ° Lifestyle Balance (1A) ° Take Charge of What’s Around You (7 or 8)	III. Mental Health and Stress ° Management of life stressors: caring for baby, feeling sad/blue, family separations, food insecurity ° Keeping calm when infant breastfeeding may be stressful/use of soothing techniques for self and baby ° Communication strategies for partner about healthy changes and family traditions ° Handling advice and critiques from others about changing behaviors
IV. Weight Loss ° Getting started with Losing Weight (1B) ° The Slippery Slope of Lifestyle Change (12)	IV. Weight Loss ° Working towards healthy weight loss through multiple strategies rather than short-term diets ° Engagement with weight loss supportive resources in community ° Breastfeeding as a means to weight loss ° Breastfeeding and importance of exclusivity and duration (12 months) for weight loss, baby’s health, and lowering of chronic disease risk ° Importance of healthy and realistic body image postpartum
V. Glucose Screening () refers to DPP Curricular Session as summarized in the Lifestyle Balance Program-DPP Lifestyle Change Program Manual of Operations 1996.	V. Glucose Screening Postpartum ° Value of getting screened for diabetes postpartum

Several additional areas were prioritized for inclusion by the RC to better reflect the realistic nature of the competing demands and underlying struggles pGDM women experienced. These topics include: support for evidence-based infant care, support for unmet mental health needs, and training related to health literacy skill building. There was consensus in the RC that in order to engage postpartum Latina mothers it would be critical to present the program as focusing less on their health and more on their roles as mothers, and specifically their role as ‘experts’ in diabetes prevention for their families. Because STAR MAMA focuses on pGDM women, the importance of breastfeeding and support for breastfeeding challenges was emphasized [5]. The RC also wanted an emphasis on social support narratives and mental health resources and to include many entry points to the vulnerability of pGDM women about unmet mental health needs that went beyond postpartum depression symptoms, such as through topics related to feelings of isolation, stress in partner or family relationships, anxiety related to having had a high-risk pregnancy and future diabetes risk, fears about financial strains, and feeling overwhelmed by infant care demands. A review of the American Academy of Pediatrics, American College of Obstetrics and Gynecologists, and American Academy of Family Physician clinical guidelines for postpartum care identified infant care, infant feeding, and maternal mental health needs as key areas requiring resource referral, reinforcing the additional content areas selected by the RC [42–44]. The RC also identified an interest in health literacy skill building content such as explaining to women how to interpret infant growth curves, read food labels, select organic or non-organic produce, and estimate the quantity of sugars in popular beverages that did not have food labels, such as *aguas frescas*.

### Steps 2 and 3. Understanding barriers and enablers for diabetes prevention among postpartum women and identifying which barriers and enablers need to be addressed.

The second and third steps in the development process were conducted concurrently and focused on an exploration of the socio-ecological factors that drive individual and family-level diabetes prevention behaviors.

### Focus groups—results about behavioral and contextual barriers and enablers and emotional truths

Women identified many types of barriers and a few enablers relevant to diabetes prevention. Table 3 summarizes examples of these barriers and enablers using the COM-B framework. Several themes emerged related to challenges in finding ways to adopt and maintain diabetes prevention behaviors in the context of their roles as mothers, wives, and the intergenerational custodians of health for their children. Several of the specific barriers that women reported are consistent with literature among other populations of pGDM women describing a multitude of challenges to diabetes prevention efforts [45, 46], such as tiredness, childcare demands, and not feeling it is ‘right’ to not be with their babies [38]. However, women in the STAR MAMA focus groups also described additional barriers, that reflected experiences related to isolation and persistent poverty associated with migration, and with some of the intergenerational barriers as described by Greenhalgh et al. [47]. However, as women also expressed pride in these roles, this was seen as an important potential enabler for the intervention framing. Examples of barriers described included finding ways to keep meals healthy when their families preferred soda and fried foods (capability), getting adequate physical activity, when they did not have motivation to get out of the house and pack up their babies for a walk (motivation), and feeling that they did not have a good idea of how to be physically active when they lived in bad neighborhoods or on busy roads (opportunity). Fears about the context of their daily lives were also apparent in several ways which impacted motivation as well as opportunity. For example, a sense of social isolation increased reluctance to try new things (motivation) and concerns about a lack of safety in their neighborhoods (physical opportunity) compounded this. The high cost of healthy foods (physical opportunity) and the inundation of poor quality yet inexpensive foods (barriers through physical opportunity) in their environments were identified—“*White bread and ‘pan dulce’ are easy to find and cheaper than whole wheat bread*” (P3-FG1)*.* “*When you are poor, you eat what you can afford*” (P2-FG3).

**Table 3:** Focus group examples of applying COM-B classifications to barriers and enablers affecting adoption of diabetes prevention behaviors

Capability	Motivation	Opportunity
An individual’s physical and psychological capacity to engage in the behavior. Includes physical capability (strength, skills, stamina) and psychological capability (knowledge, psychological skills, stamina).	Processes that affect being able to do the behavior at the relevant time and not engage in a competing behavior. Motivation is reflective (self-conscious planning) and evaluation (beliefs about what is good or bad, what will be consequences) and automatic (processes related to wants and needs, desires, reflexes and impulses).	Factors that affect the behavior in the context of the environment both physically and socially. Includes physical opportunity (time, triggers, resources, physical barriers) or social (interpersonal influences, social cues, cultural norms).
Psychological capability Knowledge: Women do not know about family friendly low sugar foods they can prepare or how to incrementally lower total sugars. Skills: Women lack navigational skills for getting physical activity in their postpartum and post-migration lives- given barriers such as unsafe neighborhoods, isolation, lack of familiarity.	Reflective motivation Beliefs: Women do not feel they are ‘supposed’ to focus on exercise for themselves in their roles as mothers (also relates to Social Opportunity, and cultural norms about what mothers can do for ‘themselves’). Outcome expectations: Women do not think their family, especially husband or partner, will go along with reductions in meat consumption, so they do not want to try and have conflict about it. *Beliefs*: Women believe that they are the cultural custodians of their family’s traditions about food, and have many ideas about how to eat healthier.	Physical opportunity Time: Women feel exhausted and have limited time when they are not caring for others to develop new approaches to preventive behaviors. Resources: Women are often food insecure and feel frustrated at the lack of options for healthy foods. *Resources*: Women are often eligible for local programs to access fresh produce that align with their desires for naturalness and healthy foods. Access: Women often do not feel they can walk or exercise near their homes which are on crowded streets or in bad neighborhoods.
Physical capability (none identified)	Automatic motivation Reactions to stress: Women often feel stressed about their lives and turn to food to comfort them, often making impulse purchases or eating large portions.	Social opportunity Social norms: Women feel pressured by other women who are experienced mothers to introduce solids and reduce breastfeeding if their babies are fussy. Cues to action: Women do not have the regular reminders they had during pregnancy to help them with diet and exercise. For most of the women, they had nutritionists or other clinic staff, such as health educators, routinely checking in and prompting women to maintain healthy behaviors. *Social support*: Women often had good experiences with the nutritionist and provider care they received in pregnancy and enjoy discussing healthy eating with others.

Italicized components are classified as enablers

Several women brought up differences before and after migration, for example, in getting physical activity. For example, before’ migration they would just walk everywhere since this was the way everyone got around (opportunity), whereas now that was difficult, as suggested by this exchange (from focus group 3):

Participant 1: “Grocery shopping is the only exercise I do. We even try to find parking very close to the store so not to have to walk too much. The story (excerpt of a woman trying to find ways to get more exercise) reminds me of this, walking to get vegetables would be a good idea for some exercise. In our countries, we used to walk.”

Participant 2: “We miss that!”

Participant 1: “Here, we go to the next block driving a car!”

There was agreement among participants that they lacked the focus they had during pregnancy to prevent diabetes and that it was critical that they engage their families in any future changes in their diet or physical activity planning. There were expressions related to the uncertainty of a clear plan but also to a loss they experienced from no longer having the substantial supports experienced during pregnancy from prenatal program case managers (motivation and social opportunity). Women described the experience of having someone who often checked on them regularly and made them feel supported and accountable in keeping up the healthy behaviors required during pregnancy, such as checking their sugar, low carbohydrate intake, and staying physically active (motivation) which women missed (motivation and capability). The following quote summarizes what participants encountered, a feeling of being un-moored in the postpartum period compared to during pregnancy: “*…after 6 weeks I delivered my baby I had my last diabetes check. And then, everything ended there because they tell you that you are OK and one remains thinking….. and now what?…*” (P3-FG1). However, women also were not as clear on how much to focus on prevention, with mixed views as to their level of T2DM risk, as with this quote: “*[after my baby] I thought: ‘Oh I don’t have diabetes anymore, let me eat a piece of bread’. Now it’s more difficult because I don’t have the ‘accountability’ that I need to be tracking what I am eating because no one will be checking it*” (P3-FG1).

After analysis and discussion of the focus group findings with the RC, we then created a conceptual model of the challenges STAR MAMA-eligible women faced in diabetes prevention (Additional file 1), which is adapted with permission from the model proposed by Greenhalgh et al. [47].

### Step 4. Determining which intervention components, including behavior change techniques and modes of delivery, could overcome the modifiable barriers identified and enhance the enablers

As a result of the types of stories evident in the focus group findings (e.g., the positive role of mothers as intergenerational custodians of family health, and the stress and sorrow of social isolation post-migration and challenges in reaching out to others for help), one of the main outcomes of Step 4 was to create first-person narratives that attempted to validate the struggle women experienced, while at the same time supporting a concrete step towards an activity that would reduce diabetes-related distress.

Further work based on the Behavior Change Wheel provided intervention function options that could be used to address the specific barriers identified in previous steps. For example, use of persuasion (such as supportive coaching or use of first-person modeling narratives) and training (such as role playing) were selected functions for overcoming barriers related to beliefs about consequences and self-efficacy, as well as addressing the perceived need for stories with direct discussion of emotional content. Additional file 2 presents a summary of the emotional truths women shared from the focus groups and of the content we developed based on it. The first column features a summary of the truth, with an example quote in column 2. Column 3 has the STAR MAMA narrative developed, and column 4 presents the associated TDF domain. As an example, focus group participants offered that one barrier to healthy eating was trying to convince their husband that more vegetables and less meat was better for reducing their risk of chronic disease, in part because this might convey they did not value the traditions any more/as much as their husbands did. Women were not sure whether a positive outcome would result from engaging in a dialogue about this topic. From an intervention development standpoint, this issue was understood as a barrier about ‘beliefs about consequences’ and ‘confidence’ or ‘self-efficacy about negotiating changes to food preparation’ (motivation and capability); the barrier was determined to be so important that it required several strategies for addressing it.

In making the STAR MAMA final components, the RC was involved in discussing what types of intervention components to include to help women overcome the barriers that were most pertinent to pGDM Latina women. For example, on the topic of ‘discussing with husband the idea to substitute vegetables for meat in traditional dishes’ we selected a peer modeling narrative of a woman discussing with her husband her idea of introducing more vegetables into their favorite recipes, which were often meat-based or meat-rich. This narrative aligned with the DPP curriculum principles of portion control and substitution of healthier for less healthy foods, while also responding to the challenges women described. These included cooking traditional recipes (for which some foods had more meat, fat, or starches than she felt was healthy) to maintain connections with family values yet also wanting to prepare new recipes with healthier ingredients so as to alter the diabetes risk for her family. The RC also focused on what type of message to use for the modified DPP content (e.g., first-person persuasive narrative, skill building content inside of the narrative) and the specific way to deliver the message (e.g., opt-in narrative, text, or part of the examples that health coaches would use for call backs).

### Step 5. Determining which policy categories could help encourage STAR MAMA content to be delivered in targeted settings

The primary policy strategy selected for the STAR MAMA program was service provision, in the form of creating a health coaching curriculum and offering it to staff, primarily at the two WIC clinics, and primary care sites where the STAR MAMA pilot would take place. By creating an opportunity to be directly involved in the delivery of intervention content, trained staff could then be called upon to help problem solve intervention delivery challenges that arose during the pilot. The 2-day training combined general health coaching skill development, such as patient-centered counseling and action planning we have applied in related work, with tailored content specific to postpartum women, including narratives developed specifically for Latinas, co-developed with experienced health coaches [48].

## Discussion

**Fig. 1:**
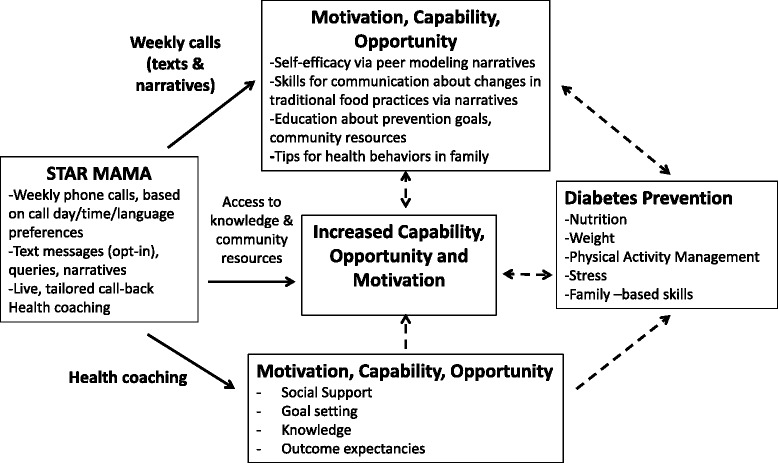
STAR MAMA intervention model for telephone-based diabetes prevention support plus supportive health coaching and linkages to resources

In its final format, STAR MAMA utilized an ATSM model to deliver diabetes prevention messages and behavior change supports through automated narrative, supportive, and educational messages combined with follow-up health coaching by trained bilingual staff and referrals to community-accessible resources (see Fig. 1 and Table 4). As shown in Fig. 1, the final STAR MAMA program includes all three areas within the COM-B model via the narratives, health coaching, and community resource sharing approaches. As shown in Table 4, beginning at 6 weeks postpartum, women receive weekly automated calls for 20 consecutive weeks. Each call is under 5 min in duration, in order to avoid overburdening women who are often holding or caring for an infant. Calls include education and tips in the form of supportive narratives and text messages about their babies and about themselves. They also include queries to participants about their behaviors, which are answered via touch tone and sent to a centralized system (e.g., “*How many times in the last 7 days have you eaten fried foods, such as tacos, chips, or French fries? Press the number of days*.”). The touch tone responses indicate either (1) a reply is ‘out of range’ based on pre-determined thresholds (e.g., has eaten fried food four or more times in the previous week); or (2) a participant has requested a call back for a given topic; these responses are in turn sent in the form of daily reports to a health coach who makes live follow-up calls to participants.

**Table 4:** Examples of STAR MAMA content—messages in the form of narratives and texted tips

		Weekly calls through STAR MAMA ATSM (weeks 1–20)																			
	DPP-relevant topic/STAR MAMA message	1	2	3	4	5	6	7	8	9	10	11	12	13	14	15	16	17	18	19	20
	Engagement with healthcare																				
	Importance of getting postpartum check-ups and obtaining medical services after delivery (i.e., blood sugar check up, HbA1c, etc.)	Q, E		Q							Q, E, F										
	Role of family planning and spacing children													E, F							E, F
	Nutrition and food insecurity																				
	Understanding your diet and options to substitute sugary foods and drinks, high fat foods with healthier options	E			E, F	N, F	E, Q			E, T				E, F	N, F	E					Q, E, F
	Understanding different nutrients like carbohydrates and sugars and their impact on your body and weight				E			E, F						E, N			E, F, T				
	How to maintain a healthy diet (i.e., even if you have to eat out at times, how to buy groceries, seek family support)			N	E	E			F, Q							N					
	Importance of continuing daily multivitamin intake even after pregnancy					E											E, F				
	Support and resources for finding community programs that offer affordable food choices												E, F							E, F	
	Understanding food labels (serving sizes, sugar and fat content, etc.)										E, F								E, F		
	Exercise and weight loss																				
	Importance of exercise and staying healthy after your pregnancy to prevent diabetes					E, Q			N	Q		Q	Q		Q, E, F	Q	Q, F	Q, F, N	Q, F		
	Resources for weight loss and exercise (i.e., text audio link for music, video, etc.)					T			N	T, E, F	T						T				X
	Tips and stories about low-cost methods to lose weight, exercise while at home or close to your community					T			N	E						N					
	Support on body image, emotional eating and health coach call back for an exercise and nutrition plan											F, N	Q, F					N	E, F		
	Stress/depression/social support/instrumental support																				
	Validation that it is normal to feel down sometimes and that both self care, reaching out to your family, and health coach support options really help	Q, F, N					E, Q	E, F				E, F				N, F					
	Accessing emotional or practical help for baby care		E, N	N			E, Q														Q, F
	Validation of experiencing family pressures after delivery and support for baby feeding and care						E													Q, F	
Child-based topics	Promoting healthy behaviors and engagement with healthcare for your infant																				
	Importance of exclusive breastfeeding for the first 6 months of child’s life + breastfeeding/bottle-feeding update	E, Q, F	E, Q, F	Q, F	E, Q, F	E, Q, F	E, N, F	E, Q, F	Q, F	Q, F	Q, F	Q, F, E	Q, F	Q, F	Q, F	Q, F	Q, F	Q, F	Q, F, E	Q, F	Q, F
	Where and who to seek support from during breastfeeding (i.e., Lactation nurse)	N, F					E, F														
	Important ways to safely bottle feed your baby and role of vitamin supplements		N	E	E	E												E, F			
	Support for understanding baby cues (including hunger cues)		N					E, F	E, F												
	Managing a fussy baby, regulating baby’s sleep environment		N	E																	
	Information on how to access online resources when your baby is sick						E, F													Q, E, T	
	Understanding why and when your baby needs immunizations and check-ups				E, Q							Q, F									
	Health coach for understanding what a baby growth chart means						E, F														

*E* education; quick tips, *N* narrative; personal anecdote or deeper information about the topic, *F* follow-up with participant through health coach call back, *Q* query; prompt to respond to specific question on health behavior(s)

STAR MAMA delivers DPP content in Spanish and English by utilizing health communication strategies that aim to: (1) validate the emotions and experiences women may struggle with in the postpartum period; (2) encourage them to integrate prevention strategies into their family life in the context of embracing their roles as intergenerational custodians of health; and (3) increase their social and material supports by facilitating access to social networks, health coaches, and community resources. The recruitment strategy focuses on community clinics with large numbers of Latina patients. Feasibility, acceptability, and health-related outcomes (e.g., weight loss, physical activity, consumption of healthy foods, breastfeeding, replacement of water for sugar sweetened drinks, and glucose screening) among STAR MAMA participants will be evaluated at 9  months postpartum using a randomized controlled trial (RCT) design. Ninety pGDM women will receive STAR MAMA call for 20 weeks beginning at 6 weeks postpartum and 90 women will receive a detailed resource guide at baseline. Women have a baseline visit, 3-month short phone survey and a 9-month postpartum follow-up survey, and will have their medical charts reviewed over the study period. The primary clinical outcomes include weight change at 9 months postpartum and receipt of recommended glucose screening postpartum. Behavioral outcomes include reported changes in adherence to DPP-recommended diet and exercise-related behaviors, duration of breastfeeding, and changes in public health literacy-associated skills.

This study has a number of strengths, primarily the close involvement of the Regional Group of stakeholders in all phases of the development and pilot testing of the STAR MAMA calls and health coaching materials, and the use of a comprehensive theory-informed approach, the COM-B and BCW, which enables a more detailed and thorough planning process [49]. As well, the extensive use of focus group transcripts in creating narratives to address ‘emotional truths’ and for forming health coaching guides are additional strengths. There are however, several weaknesses, including that the focus groups may not have reached some of the most at-risk women who were not able to travel to the sites, due to important opportunity-related barriers, such as transportation, or for those that can affect motivation, such as depression or stress. We tried to alleviate this potential problem by incorporating the input of the providers across the continuum of prenatal and postpartum care services, to help identify topics that would be most pertinent to women who were not represented in the focus group data. Another limitation of the study is that many of the barriers women faced were related to the high rates of poverty they experienced on a daily basis, and the STAR MAMA program was not able to provide substantial food or other service vouchers to aid with the poverty-related barriers that impact health. We tried to address this issue by connecting women to free community programs and services, as with our closely working with the Women, Infant, and Children’s (WIC) programs in both communities. Finally, the component of STAR MAMA that relies on the telemedicine in the form of outgoing calls and data reports sent to providers, requires a substantial commitment to learning and maintaining the system infrastructure which may provide challenges for programs who wish to take on this model.

## Conclusions

Here, we present the participatory engagement methods, development of the conceptual framework, intervention development steps and the final tailored content for an adapted DPP program tailored for a high-risk population. Because Spanish-speaking women comprise a large proportion of the STAR MAMA target population in California, several aspects of the development process focus on input from Latina pGDM women, their providers, and theoretical considerations related to adoption of preventive health behaviors that have been found to be congruent with Latina health beliefs and communication preferences.

## Additional files

Additional file 1: Levels of Influence Affecting Focus Group Participants. (PPTX 93 kb)

Additional file 2: Analysis of Focus Group Emotional Truths, Corresponding TDF Category and Examples of Related STAR MAMA Narratives. (DOCX 32 kb)

## Abbreviations

ATSM: Automated Telephone Self-Management Support, which is a Health IT telephone-based program, involves a combination of weekly calls with recorded content as queries, narratives, and texted ‘tips’ and resources, and ‘live’ coach call backs for health counseling in specific target areas, based on responses
pGDM: Postpartum women who recently had a clinical diagnosis of gestational diabetes during pregnancy
STAR MAMA: Support via Telephone Advice and Resources/Sistema Telefónico de Apoyo y Recoursos-MAMA
