# Supplementary material for: Applying the COM-B model to creation of an IT-enabled health coaching and resource linkage program for low-income Latina moms with recent gestational diabetes: the STAR MAMA program
Source: Implement Sci. 2016 May 18;11:73. doi: 10.1186/s13012-016-0426-2 (PMC4870786; doi:10.1186/s13012-016-0426-2)
Supplement: Supplementary file 1 — Levels of Influence Affecting Focus Group Participants. (PPTX 93 kb) [file 13012_2016_426_MOESM1_ESM.pptx]

## Slide 1
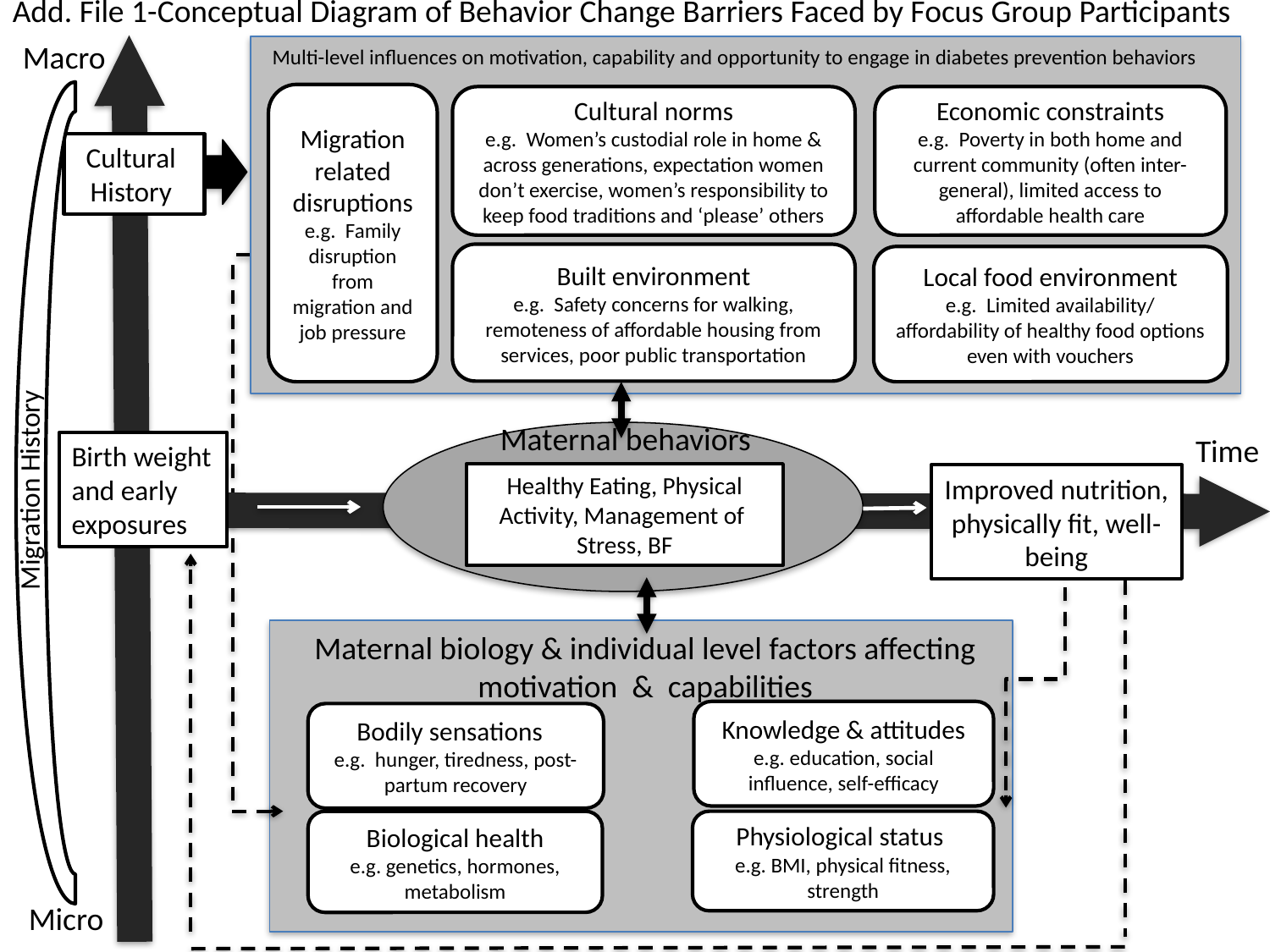

Add. File 1-Conceptual Diagram of Behavior Change Barriers Faced by Focus Group Participants
Macro
Multi-level influences on motivation, capability and opportunity to engage in diabetes prevention behaviors
Migration related disruptions
e.g. Family disruption from migration and job pressure
Economic constraints
e.g. Poverty in both home and current community (often inter-general), limited access to affordable health care
Cultural norms
e.g. Women’s custodial role in home & across generations, expectation women don’t exercise, women’s responsibility to keep food traditions and ‘please’ others
Cultural
History
Built environment
e.g. Safety concerns for walking, remoteness of affordable housing from services, poor public transportation
Local food environment
e.g. Limited availability/ affordability of healthy food options even with vouchers
Maternal behaviors
Time
Birth weight and early exposures
Migration History
Healthy Eating, Physical Activity, Management of Stress, BF
Improved nutrition, physically fit, well-being
Maternal biology & individual level factors affecting motivation & capabilities
Knowledge & attitudes
e.g. education, social influence, self-efficacy
Bodily sensations
e.g. hunger, tiredness, post-partum recovery
Biological health
e.g. genetics, hormones, metabolism
Physiological status
e.g. BMI, physical fitness, strength
Micro
