# Supplementary material for: Applying the COM-B model to creation of an IT-enabled health coaching and resource linkage program for low-income Latina moms with recent gestational diabetes: the STAR MAMA program
Source: Implement Sci. 2016 May 18;11:73. doi: 10.1186/s13012-016-0426-2 (PMC4870786; doi:10.1186/s13012-016-0426-2)
Supplement: Supplementary file 2 — Analysis of Focus Group Emotional Truths, Corresponding TDF Category and Examples of Related STAR MAMA Narratives. (DOCX 32 kb) [file 13012_2016_426_MOESM2_ESM.docx]

Add. File 2. Emotional Truths, Corresponding TDF Category and Examples of STAR MAMA Content Focusing on Truths

| **NEGATIVE EMOTIONAL TRUTHS** |  | **EXAMPLES FROM FOCUS GROUPS (Participant# - Focus Group#)** |  | **STAR MAMA NARRATIVES** | **TDF Category** |
| --- | --- | --- | --- | --- | --- |
| **Women are afraid they cannot live up to their own expectations that they can make their family healthy, in contrast to how they felt when they were pregnant.**   - Women are worried they are not doing enough for their children already and feel they don’t have good habits to help their kids. - Women want the whole family to be engaged in the changes, so they will be supported, but often are not confident they know how to make this happen. - Women don’t want to disappoint others -they want to ‘justify’ these choices. |  | *“…I imagine what an ideal mother would be. I imagine someone waking up at 7 in the morning and just doing all these things. Going out by 10, you are at the park already and had been playing for 20 minutes. For me, I’ve gotten into a bad routine…” (P1-FG2)*  *“[The hardest part] is to do things not because someone will recognize you, not because someone will tell you how good you look but because you have to think that you have children and if I get sick I’ll be a problem for them and I wont be able to help them.” (P1-FG1)*  *“My husband says to me’ how are you going to teach them if you are not doing that yourself?” (P3-FG1)*  *Women in my family are very strong and we have a lot of pride in our family, we don’t ask for help.” (P3-FG2)*  *“But I always try to find natural foods first.” (P4-FG1)*  *“but I don’t want to sacrifice my daughters” [by making them change their diets to avoid sodas or large amounts of sugars and carbs], so I was getting the healthiest food I could” (P4-FG1)* |  | *Many women who helped create the STAR-MAMA program told us that keeping healthy was easier when they were pregnant since focusing on a healthy baby was an important goal. You may not realize it but your baby gave you a gift by having a hard pregnancy, since now you know how to focus, even if it seems harder now. Listen to Georgina’s story:*  *Georgina had been careful about her diet when she was pregnant but now she worried she’d fall into her old eating habits, not lose weight, and stay at risk for diabetes. She admitted to herself that even though she loved to cook traditional dishes many of them had too much meat. Her doctor had told her that her baby should grow up with a healthy diet, and less meat. This made Georgina feel that now was the time to learn to make lasting changes. She told her husband that she wanted to make some changes in the foods they ate, so their baby would grow up eating more of the healthy foods that were part of their families’ traditions, and he agreed to try them. Georgina combined one of her mother’s flavorful salsas with a larger amount of vegetables and a smaller portion of meat. She and her husband agreed the changes tasted fine. Georgina was happy she had spoken to her husband about their family meals.”*  *Story models engaging husband and justifying changes within healthy family traditions | *Motivation and Goals; Beliefs about Consequences; Social Influences; Social Role and Identity* |
| **Women miss external motivators ‘pressuring’ them to be healthy since this gave them self-efficacy to be ‘good’ for the baby. Now, they are uncertain how to develop self-esteem and independence to make prevention important in their daily lives.**  On the one hand they want to be kept ‘in line’ re their diabetes risk, but also recognize they often feel alone in this work and want help from others.  They liked having someone look after them during pregnancy but they also say they need to learn more independence, and through independence they can feel intrinsically “pressured” or motivated. |  | *“It is difficult after you have had someone for some time telling you what to do and suddenly you don’t have it anymore and you may feel like you had been left out in the air (alone). I know that nobody will tell me ‘ oh, you cant eat this or that’- I don’t have the accountability that I need to be tracking what I eat. And that is not so good.“ (P3-FG1)*  *Exchange: (from Focus Group 1)*  *Participant 1; “I don’t understand what is happening to us. I don’t know if it is part of a problem with our self-esteem. It might be because I am sure that all of us know what is good for us and what is bad for us but we don’t do it. And I don’t know why we can’t do anything for ourselves.*  *Facilitator: is it because you are too busy?*  *Participant 4: “I don’t think so. There is always time to say oh its time to get the children. I have to do the laundry but we don’t make time to do things for us”*  *1: “But if we wanted to we could have time for ourselves.”*  *4: “Yes, because we have time to watch telenovelas.”*  *1; “Yes, we did eat healthy and went for a walk in the neighborhood everyday. “*  *4:”We were doing it as an obligation. “*  *1: “Because we were feeling the pressure.”*  *4: “My doctor weighs me and tells me I am not following the rules and if you don’t take care of yourself no one else will do it for you.” -1*  *1: “Yes, that’s another important thing; we don’t have anybody else more than ourselves.*  *“But I followed it a lot (a plan) when I was pregnant, but now its more difficult because you get comfortable, so I say, ‘I’ll eat this’ and well its because I don’t have that pressure anymore” (P2-PG4)* |  | *Patricia: “ I was struggling a lot with my feelings after having my baby and even though my doctor had told me to exercise, I just couldn’t find the energy to do more!! I was overwhelmed and worried about all the things I needed to do. When my health coach called me right after I joined the Star-Mama program, I mentioned this to her and she helped me find a mom’s group where I could meet new moms like me, talk and share experiences. I joined a group and it has been so good for me!—I can talk about my worries and hear points of views that are helpful to me. I also met a new mom who lives close by and we are planning to take walks together and watch each other’s children in our homes so we can relax when we are having a hard day. I think we will be able to help each other and I am looking forward to that.”*  *Story shows a woman’s enablement to find support without ‘asking for help’, and to find others to look out for each other with. – | *Motivation and Goals; Social Influences; Environmental Context and Resources* |
|  |  |  |  |  |  |
| **Women are not sure how to translate prevention behaviors from their lives before immigration to now, especially ‘in public’ and for exercise this is a big challenge.** |  | *Exchange: (from Focus Group 3)*  *Participant 1:“Grocery shopping is the only exercise I do. We even try to find parking very close to the store so not to have to walk too much. The story (excerpt of a woman trying to find ways to get more exercise) reminds me of this, walking to get vegetables would be a good idea for some exercise. In our countries, we used to walk.”*  *2- “We miss that!”*  *1: “Here, we go to the next block driving a car!”*  *2: “Every time I go back to my home in Mexico, I walk everywhere even though we have a car. I always try to walk around the town even if people think I’m crazy. Here, I try to park away from the entrances so I can walk.”*  *“There are many like me who don’t have that ( a daughter who walks with her mom/does zumba classes). That’s why I feel so sad because when I tell her to go for a walk or do exercise, she just doesn’t want to go. And we’re the models for them. So I say, Dios mio- I want to be different.” (P1-FG1)* |  | *Ana: “ Believe me, it wasn’t easy! Finding the time to exercise was my greatest challenge, after having my baby I gained almost 60 pounds and knowing that diabetes runs in my family I just wanted to change whatever I could to be healthier--- but I also knew wishing was not going to change anything! I had to start somewhere so I decided to start easy and just try to get moving by walking with the baby stroller. I had not realized how much I had gotten used to the car, and there is a market just a half a mile from my house. If I walked there a couple times a week, I could put my purchases underneath the stroller, and avoid loading and unloading the baby in the car seat, which took a lot of time anyway. It has been a slow process to lose the pounds but I am feeling much better, losing those first pounds already and little by little I have been adding more minutes to my walking.”*  *Story validates that it is a challenge to get exercise and also models a new skill (walking to market with baby stroller) and increasing motivation by encouraging action planning through small increments of change with minutes walked. | *Behavioral Regulation; Motivation and Goals; Skills* |
| **POSITIVE EMOTIONAL TRUTHS** |  |  |  |  |  |
| **Women are proud of their influence and responsibility -for their families.**  Women self-identify as the one her children will look to for learning how to make choices. The choices they see are something she controls. The positive behaviors she exhibits could benefit her, but ultimately they are for the good of her family. |  | *“ I couldn’t tell my children to not drink soda if I am drinking it. We have to lead by example. Now, there is no soda in my house. We all drink water.” (P3-FG3)*  *“For me it was difficult because I have children so we changed their diet too.” (P3-FG1)*  *“What I did with my family is to say everybody is eating the same. Everybody will eat what I eat. This is not a restaurant.. And because they [the family] saw how I was struggling, they know what I went through. So they learned.”(P4-FG1)*  *“Since I cook for everybody what I did was to adjust the amounts of what I ate. So I didn’t need to sacrifice my family. We used to buy sodas for everyone at home but I didn’t drink it. So, little by little my family learned that sodas are not good for you and everybody started drinking less soda. At the end of my pregnancy nobody was drinking soda and we didn’t but it anymore.” (P4-FG1)*  *“Every time I have my appointment I am very punctual because I worry about my health. I want to take care of myself more because I want to be well for my daughter and the people that love me. (P3-FG4)* |  | *Veronica: “I was proud to bring more fresh fruits and vegetables into my family’s meals. But when I started looking around more at the produce at the market I noticed there were lots of signs about organic produce and I wondered if this organic produce was worth the price? I had heard that organic fruits and vegetables were better since they didn’t have chemicals but then, why did they cost more if they had less added to them? I talked to my health coach and she explained how the farmers grew organic and non-organic produce differently, and she also explained which fruits and vegetables absorbed the most chemicals. There is a good list of which fruits and vegetables to try to buy organic when they were affordable. Here is the best if organic list-- strawberries, peaches, spinach, bell peppers, and apples – since these foods all absorb chemicals easily; while bananas, cantaloupes, sweet corn, mango, papaya, avocados, onions and asparagus don't.. So now, when I go to the market I look at how to use this information to make choices because I’ve learned how to select my produce!”*  Story models pride in learning new information to improve family’s access to affordable healthy food. | *Social Role and Identity; Env. Context and Resources; Knowledge* |
| **Women experience personal growth and pride through developing new skills, sharing them with others, and seeing the impact across generations.**  Developing new skills brings self-satisfaction that is unique to post-migration life where fewer family members are around for support.  Empowerment through knowledge can counteract some of the fatalistic views they hear and views they don’t agree with about their baby care. |  | *“When I came here I only came with my husband. I don’t have any relatives here and when my son was born I didn’t know how to drive and I had troubles going to my appointments, to my classes and other things and I was feeling depressed. And I told my husband, you teach me to drive or I will take the care and learn by myself’ and he taught me. And now I go on my own to my appointments or to the super or the laundry and I feel more independent. Before, I was spending all my time thinking about how my family was doing in my country. Now I am busy. I am more motivated because I can do more things and I don’t have to wait for someone all the time to give me a ride to bring me to work or spend lots of time on buses. I feel calmer.”(P2-FG1)*  *“I have a friend who tells me my refrigerator is boring because I only have fruits and vegetables. They get surprised when my children like vegetables. I don’t have any juices. I think nutrition is very important since they are very young because that is what they are going to give to the next generations.”(P6-FG3)*  *“My mother in law was always complaining about feeling ill. Once we brought her to get checkups- she has diabetes. However, now it is under control because I made a lot of changes in her diet. Even the doctor asked what she was doing- I told him she was eating better.”(P2-FG3)*  *“I was embarrassed (when I did not want to do as my mom and grandmother instructed me about feeding my baby and how much I should be eating) because they wanted to help me and keep me well nourished. When they (visited the doctor) they learned too, because they didn’t know it is not only give and give.”(P7-FG3)*  *“We have to make decisions for ourselves. For example, my sister comes to visit me and says, ‘just eat, at the end we are all going to die of something’. I tell her ‘yes’ but if I can delay death a little bit, that’s better. Then she gets mad at me because she tries to give gummy bears to my kid and I tell her ‘no’. So I think it is important to make the decision and to learn what to feed your children. “(P3-FG3)*  *We ask advice to our moms; my mom, doctor, and family. But it is true; you are not going to do everything they tell you. You take 3 or 4 opinions and then you decide what you agree with and what you don’t agree with. (P1-FG4)* |  | *“Rosa was big on sweets and loved pancakes or frozen waffles especially in the mornings. She spoke to her doctor about her cravings because she wanted to keep her weight on track. Listen to Rosa's story: I really like sweets and loved pancakes and frozen waffles especially in the mornings. When I spoke with my doctor about my cravings and that I wanted to keep my weight on track. He told me to check how many calories were in them.  I did and I learned that three pancakes were more calories than I wanted, but two were ok, and one waffle was even better than two.  I wanted to feel satisfied so I tried having less pancakes and add fresh fruit and a little bit of nuts–, this tasted great and was filling.  I feel proud of myself for cutting down the calories and still being able to eat what I liked. I'm planning to make this kind of changes, little by little by little.“*  *Story shows pride in developing a new skill and that it is ok to ask for/receive advice, since you can make it into your OWN plan. | *Behavioral Regulation; Beliefs about Capabilities; Motivation and Goals* |
